# Thrombus aspiration catheter improve the myocardial reperfusion of STEMI patients with high thrombus load during the emergency PCI operation

**DOI:** 10.1186/s13019-019-0974-z

**Published:** 2019-09-23

**Authors:** Ping Li, Jiang-Wen Ruan, Ming Liu, Si-Yao Li, Zheng-Dong Wang, Wen-Chao Xie

**Affiliations:** Department of Cardiology, Yulin First People’s Hospital (The Sixth Affiliated Hospital of Guangxi Medical University), No.495 Education Middle Road, Yuzhou District, Yulin, 537000 Guangxi China

**Keywords:** Thrombus catheter aspiration, Balloon dilatation, High thrombus load, Acute ST-elevation myocardial infarction (STEMI), Percutaneous coronary intervention (PCI), Major adverse cardiac events (MACE)

## Abstract

**Objective:**

This study aims to discuss the efficacy and safety of the application of thrombus aspiration catheters during emergency PCI operations for acute ST-elevation myocardial infarction (STEMI) patients with high thrombus load.

**Methods:**

A total of 204 patients diagnosed with acute STEMI and high thrombus load in the Sixth Affiliated Hospital of Guangxi Medical University from July 1, 2016 to June 30, 2017 were selected for the present study. These patients were randomly divided into two groups: thrombus catheter aspiration group (group A, *n* = 101), and balloon dilatation group (group B, *n* = 103). The blood flow of the culprit coronary artery in the thrombolysis in myocardial infarction (TIMI) immediately after the emergency PCI operation in these two groups of patients was recorded. Then, an echocardiogram was performed to determine the left ventricular end-diastolic diameter (LVEDD) and left ventricular ejection fraction (LVEF) after the operation, and data on major adverse cardiovascular events (MACE) during the 30 days of postoperative follow-up were collected.

**Results:**

The comparative difference between these two groups of patients in terms of hypertension, smoking, diabetes, usage rate of GPIIb/IIIa receptor antagonist, time from hospitalization to balloon dilatation (D2B) and other basic clinical data was not statistically significant (*P* > 0.05). The postoperative TIMI flow grade of these two groups of patients improved, and the comparative difference between the data obtained from these two groups was statistically significant (*P* < 0.05). The comparative difference between these two groups in terms of LVEDD and LVEF at 7 days after the operation was not statistically significant (*P* > 0.05). There was a difference in the occurrence rate of MACE in these two groups of patients during the 30 days of postoperative follow-up, but the comparative difference between these two groups was not statistically significant (*P* = 0.335).

**Conclusion:**

The application of thrombus aspiration catheter during the emergency PCI operation of STEMI patients with high thrombus load can better improve the myocardial reperfusion. There is no basis for increasing the stroke occurrence risk. However, it obviously fails to improve the recent prognosis and more studies need to explore its effect on myocardial remodeling and major adverse cardiovascular events.

## Introduction

ST-elevation myocardial infarction (STEMI) is one of the most common acute and serious cardiovascular diseases, and has become one of the main causes of sudden death in adults [1]. It is reported that the formation of thrombus is one of the first and foremost pathogenesis for all acute coronary syndromes (ACS) [2]. Percutaneous coronary intervention (PCI) is presently the internationally recognized preferred strategy for the treatment of STEMI. Since a traditional PCI operation may cause the formation and detachment of small thrombus, and lead to no-reflow or slow-reflow of the culprit vessel, and poor myocardial perfusion can be observed in 20–40% of patients, this would have an adverse impact on long-term survival [3–5]. Thus, the thrombus removal device emerges at the right moment. There are numerous studies on thrombus removal devices at home and abroad, but its safety and efficacy failed to reach a consensus continuously. Especially for the thrombus aspiration catheter, the results of several small random clinical tests have shown that the thrombus aspiration catheter can improve myocardial perfusion, but has no significant impact on the clinical prognosis [6–8]. But many studies have failed to reach an agreement on whether the thrombus aspiration catheter increases the death rate of patients and stroke occurrence rate [9–11]. The present study aimed to further discuss the recent clinical prognosis of the thrombus aspiration catheter for STEMI patients with high thrombus load by observing the impact of the application of a thrombus aspiration catheter during the emergency PCI operation of STEMI patients with high thrombus load upon myocardial reperfusion and cardiac function.

## Materials and methods

### Study object

Patients diagnosed with STEMI and high thrombus load in the Sixth Affiliated Hospital of Guangxi Medical University from July 1, 2016 to June 30, 2017 were selected for the present study. The study plan was implemented after the approval of the Medical Ethics Committee of the hospital. All patients were informed of the study and provided a signed informed consent.

### Test design

All selected patients are diagnosed with STEMI and high thrombus load via coronary angiography, and their selection conformed to the standards. These patients were divided into two groups, according to the hospitalization order and random number table: thrombus catheter aspiration group (group A), and balloon dilatation group (group B). Before the emergency interventional therapy, these two groups of patients were given a load dosage of clopidogrel (300 mg) and aspirin (300 mg) for impact therapy, and orally received clopidogrel (75 mg) and aspirin (100 mg) once a day after the operation. The Seldinger method was used for the approach on all patients through the right radial artery or femoral artery. It was verified by coronary angiography that the infarct-related artery (IRA) was the high thrombus load, and patients with multi-vessel lesion were only treated for the main culprit vessel. In group A, an Export AP thrombus aspiration catheter is used for repeated aspiration for 3–5 times. If the stent cannot be directly implanted in the narrow part of the IRA lesion, a balloon dilatation can be carried out before implantation. In group B, the stent is directly implanted after the balloon dilatation, and the number of times the guide wire was passed through the narrow part of the lesion, as well as the number of times of balloon dilatation, was reduced as much as possible in this process. Coronary angiography was carried out after the stent was implanted. If the culprit coronary artery had no-reflow, the patient was treated by injecting 10–12 ml of tirofiban hydrochloride through the coronary artery, and the determination time of final angiographic results was 1 min later. The above operation was carried out by two or more physicians with rich intervention experience.

### Result observation

#### Recording of thrombolysis in myocardial infarction (TIMI) flow grade before and after the PCI operation

##### Observance of major adverse cardiovascular events (MACE) during the 30 day postoperative follow-up (outpatient service and telephone)

The MACE included cardiac death, stroke, nonfatal myocardial infarction, and target vessel revascularization (new PCI or CABG).

### Statistics processing

The SPSS 17.0 statistical software was used for the data analysis in the present study. *P* < 0.05 was considered statistically significant.

## Study result

### Selection results and basic clinical data of the two groups of patients

A total of 204 STEMI patients with high thrombus load conformed to the inclusion criteria. These patients were divided into two groups, according to the hospitalization order and random number table: group A (*n* = 101), and group B (*n* = 103). There was no significant difference in basic clinical data of hypertension, smoking, diabetes mellitus, usage rate of GPIIb/IIIa receptor antagonist and D2B between the two groups.(*P* > 0.05, Table 1).

**Table 1:** Comparison of two groups of patients with basic information

Items/ groups	Thrombus catheter aspiration group (*n* = 101)	Balloon dilatation group (*n* = 103)	*P* value
Age (years old, $\overline{x}$ ± *s*)	60.94 ± 11.417	62.14 ± 10.775	0.443
Sex (n, %)			0.808
Male	78 (77.2)	81 (76.8)	
Female	23 (22.8)	22 (21.4)	
Hypertension (n, %)	64 (63.4)	63 (61.2)	0.746
Diabetes(n, %)	13 (9.7)	10 (9.7)	0.475
Smoking history (n, %)	59 (58.4)	58 (56.3)	0.761
Cardiac function (Killip grading) (n, %)			0.897
1 level	86 (85.1)	89 (86.4)	
2 level	7 (6.9)	7 (6.8)	
3 level	1 (1.0)	2 (1.9)	
4 level	7 (6.9)	5 (4.9)	
GPIIbIIIa receptor antagonist (n, %)	14 (13.9)	16 (15.5)	0.736
Culprit coronary artery (n, %)			0.411
LM	1 (1.0)	0	
LAD	58 (57.4)	61 (59.2)	
LCX	7 (6.9)	12 (11.7)	
RCA	35 (34.7)	30 (29.1)	
The number of vessels suffering disease (n,%)			0.718
Single vessel	38 (37.6)	41 (39.8)	
Double vessels	44 (43.6)	47 (45.6)	
Triple vessels	19 (18.8)	15 (14.6)	
D2B(min,$\overline{x}$ ± *s*)	88.41 ± 19.208	87.82 ± 15.625	0.814
From onset to D2B(h,$\overline{x}$ ± *s*)	7.02 ± 2.958	7.67 ± 2.697	1.040

Note: *LM* left main, *LAD* left anterior descending, *LCX* left circumflex, *RCA* right coronary artery, *D2B* time from hospitalization to balloon dilatation

### Comparison of these two groups of patients in terms of coronary blood flow TIMI grade and no-reflow occurrence rate after the operation

TIMI blood flow after the PCI operation of these two groups of patients: in terms of TIMI grade 0 and grade 1 blood flow, there were no patients in both groups; in terms of TIMI grade 2 blood flow, there were five patients in group A and 14 patients in group B; in terms of TIMI grade 3 blood flow, there were 96 patients in group A and 89 patients in group B; in terms of no-reflow: there were five patients in group A and 14 patients in group B. There was significant difference in theTIMI blood flow between the two groups (5.0% vs. 13.6%, *P* = 0.034; Table 2).

**Table 2:** Comparison of TIMI blood flow in infarct-related artery after emergency PCI

TIMI flow grades/ groups	A group (*n* = 101)	B group (*n* = 103)	χ^2^	*P* value
TIMI 0 level (n, %)	0 (0)	0 (0)		
TIMI 1 level (n, %)	0 (0)	0 (0)		
TIMI 2 level (n, %)	5 (5.0)	14 (13.6)		
TIMI 3 level (n, %)	96 (95.0)	89 (86.4)		
NO-reflow (n,%)	5 (5.0)	14 (13.6)	4.509	0.034

Note: *PCI* percutaneous coronary intervention, *TIMI* thrombolysis in myocardial infarction, A Thrombus catheter aspiration, *B* Balloon dilatation

### Comparison of left ventricular end-diastolic diameter (LVEDD, mm) and left ventricular ejection fraction (LVEF, %) at seven days after the operation between the two groups of patients

The LVEDD and LVEF in group A were higher than those in group B. However, the data of these two groups were within the range of the normal value, and there was significant difference in LVEDD and LVEF between the two groups (*P* > 0.05, Table 3).

**Table 3:** Comparison of LVEDD and LVEF at 1 week after emergency PCI

Items/ groups	A group (*n* = 101)	B group (*n* = 103)	*P* value
LVEDD (mm)	44.72 ± 4.911	44.34 ± 4.994	0.589
LVEF (%)	53.03 ± 9.648	51.62 ± 9.537	0.304

Note: A thrombus catheter aspiration. *B* Balloon dilatation, *PCI* percutaneous coronary intervention, *LVEDD* left ventricular end-diastolic dimension, *LVEF* left ventricular ejection fraction

### Comparison of MACE within 30 days between the two groups of patients

There are seven patients with MACE in group A, and all were had cardiac death, including five patients with heart failure, one patient with ventricular fibrillation, and one patient with sudden death. There are four patients with MACE in group B, and all had cardiac death, including two patients with heart failure and two patients with ventricular fibrillation. There was no stroke incidences in these two groups, and the comparative difference between these two groups in terms of total MACE occurrence rate was not statistically significant (6.9% vs. 3.9%, *P* = 0.335; Table 4).

**Table 4:** Comparison of the incidence of MACE in both groups

Events/ groups	A group(*n* = 101)	B group (*n* = 103)	χ^2^	*P* value
Cardiac death (n, %)	7 (6.9)	4 (3.9)		
Target lesion revascularization (n, %)	0	0		
Non-fatal myocardial infarction (n, %)	0	0		
cerebral ischemic stroke (n, %)	0	0		
Total MACE (n, %)	7 (6.9)	4 (3.9)	0.928	0.335

Note: A thrombus catheter aspiration. *B* Balloon dilatation, *MACE* major adverse cardiovascular events

## Discussion

The occurrence rate of myocardial no-reflow in patients undergoing an emergency PCI operation remains within 20–37%, particularly for STEMI patients with high thrombus load. Hence, this would seriously affect the prognosis of patients [12]. The reason for the slow flow and even no-reflow of the culprit vessel after the STIMI patient undergoes an emergency PCI operation remains unclear, and involves multiple factors. During the PCI operation, the operation may lead to the thrombus detachment, and form the move of small thrombus towards the distal vessel. Hence, the microthrombosis of the distal vessel is the main reason for the occurrence of no-reflow [13]. The TAPAS study was a single-center randomized study that used a blind spot to assess the endpoint, covering 1071 patients [9]. These patients were divided into two groups: traditional PCI operation group and thrombus aspiration group. It was found that the myocardial blush grade (MBG) of the thrombus aspiration group was superior to that of the traditional PCI operation group (*P* = 0.032) through the follow-up within 30 days to 1 year after the operation. The MUSTELA study carried out by Marco D C et al. [14] in 2012 also obtained similar results to the TAPAS study. In the present study, five patients had no-reflow in group A after the PCI operation, while 14 patients had no-reflow in group B, and the comparative difference between groups was statistically significant (95.0% vs. 86.4%, *P* = 0.034). These results indicate that the thrombus aspiration catheter can effectively improve the myocardial reperfusion.

The persistent occlusion of the distal microvessel would lead to cardiac remodeling and changes in cardiac structure [15]. The left ventricular remodeling was characterized by the gradual increase in end-diastolic volume. The study results of De Luca et al. [16] revealed that the end-diastolic volume and systolic left ventricular volume of patients in the thrombus aspiration group were significantly lower than those of patients in the common PCI group at 6 months after the operation. The LVEDD and LVEF of these two groups at 7 days after the operation were different, but the data comparative difference of these two groups was not statistically significant (*P* > 0.05). Since the myocardial remodeling was affected by many factors, this can be correlated to the soluble medium secreted by the renin-angiotensin system. Due to the temporal correlation, the myocardial remodeling cannot lead to the obvious change in cardiac structure within a short time after the operation. Even though the same experienced cardiac color ultrasound examination physician would be required to carry out the operation, it still cannot completely eliminate the interference of subjective factors. Therefore, it still cannot be concluded from the study results whether these two groups of patients have no difference in cardiac remodeling after the PCI operation. The follow-up and cardiac color ultrasound examination time should be properly extended, and the examination should be carried out for many times.

The ability of thrombus catheter aspiration to improve the myocardial perfusion has been recognized, but a consistent conclusion on whether it can improve the survival prognosis of STEMI patients could not be obtained. Prior studies have suggested the death rate in the thrombus aspiration group was lower [17, 18]. However, the data comparative difference between these two groups was not statistically significant (*P* = 0.57). Furthermore, the difference in the total occurrence rate of MACE in these two groups of patients was also not statistically significant (*P* = 0.48). Another prospective random study, TOTAL test [10], also obtained results that were similar results to the TAPAS test. However, the TOTAL test found another problem: The stroke occurrence rate of the experimental group was higher than that of the control group (*P* = 0.015) after 1 year of follow-up. A TOTAL sub-study [19] conducted an analysis on this problem. It was found that 33 patients and 16 patients suffered from stroke within 30 days after the operation in the experimental group and control group, respectively, and the data comparative difference between these two groups was statistically significant (*P* = 0.015). The investigator found another more interesting phenomenon through the segmentation of time nodes. The stroke occurrence rate was the highest within 48 h after the operation. The difference in occurrence rate in these two groups was statistically significant (*P* = 0.025), while the difference in stroke occurrence rate in other time ranges was not statistically significant. The total stroke occurrence rate for the experimental group was higher than that for the control group within 180 days after the operation, and the data comparative difference of these two groups was statistically significant (*P* = 0.03). The investigator concluded that the reason for the higher stroke occurrence rate in the thrombus aspiration group may be that the small thrombus adhered to the aspiration catheter in the thrombus aspiration process, and flowed from the culprit vessel to the entire vascular system. Attention should be given to a fact: the stroke does not just occur during the perioperative period. It is presumed that the increased risk of stroke is due to thrombus aspiration catheters during surgery, which seems to lack evidence. A meta-analysis released in 2015 concluded that the thrombus aspiration catheter does not increase the occurrence rate of stroke [20]. The occurrence rate of MACE in group A was higher than that of in group B within 30 days after the operation in the present study, but the data comparative difference of these two groups was not statistically significant (*P* = 0.335), and these two groups of patients did not suffer from stroke. The observation results of stroke in the present study were different from that in the TOTAL study. On one hand, it may be due to different levels of operation. Hence, the small thrombus that detached during the operation would flow from the lesion part of the culprit vessel to the ascending aorta along the aspiration catheter, and flow to the entire vascular system. On the other hand, it may be correlated with the type of aspiration catheter. Some aspiration catheters are of the side-hole type. The negative pressure of various side holes would uneven in the aspiration process, which would very likely lead to the detachment of small thrombus. The suction tube used in this study is end-hole type and has good negative pressure sealing. In addition, this can reduce the separation of small thrombus during surgery, which may be related to stroke not observed in this study.

## Conclusions

In summary, the thrombus aspiration catheter can improve the myocardial reperfusion of the STEMI patient with high thrombus load. Although the short-term prognosis failed to obtain a benefit, the stroke risk did not increase. Further research is needed on myocardial remodeling and its impact on major adverse cardiovascular events. Since the follow-up time of the study was shorter, and the long-term prognosis failed to be further verified, more clinical tests and longer follow-up observations would be required for performing the analysis.

## Data Availability

The datasets used and/or analysed during the current study available from the corresponding author on reasonable request.

## Abbreviations

ACS: acute coronary syndromes
IRA: infarct-related artery
LVEDD: left ventricular end-diastolic diameter
LVEF: left ventricular ejection fraction
MACE: major adverse cardiovascular events
MACE: major adverse cardiovascular events
MBG: myocardial blush grade
PCI: Percutaneous coronary intervention
STEMI: ST-elevation myocardial infarction
TIMI: thrombolysis in myocardial infarction
TIMI: thrombolysis in myocardial infarction
